# Nuclear Medicine Imaging of Gynecological Malignancies: The Tumor, the Tumor Microenvironment, and Beyond

**DOI:** 10.1055/s-0044-1787806

**Published:** 2024-06-24

**Authors:** Kgomotso M.G. Mokoala, Michael M. Sathekge

**Affiliations:** 1 Department of Nuclear Medicine, University of Pretoria and Steve Biko Academic Hospital, Pretoria, South Africa; 2 Nuclear Medicine Research Infrastructure (NuMeRI), Pretoria, South Africa

Gynecological malignancies represent a significant burden on women's health worldwide. While cervical cancer has screening tests, the remainder of the gynecological malignancies do not have programs for early detection, and as such patients usually present late with advanced disease and limited treatment options. The advancements in nuclear medicine in the form of improved instrumentation including hybrid imaging as well as production of innovative diagnostic and therapeutic radiopharmaceuticals offer insights into the tumor itself, its microenvironment, and beyond. Considering precision medicine, this offers hope for tailored therapeutic strategies for cancer patients. This editorial explores radionuclide imaging, delving into its applications in tumor assessment (staging, response assessment, and suspected recurrence), assessing the tumor microenvironment, and extending into novel horizons for comprehensive management of gynecological cancers.

Positron emission tomography (PET) with [ ^18^ F]F-fluorodeoxyglucose ([ ^18^ F]F-FDG), which exploits the cancer cells increased glucose metabolism, has remained the backbone in the diagnostic workup of gynecological malignancies. In a comprehensive review by Khessib et al, the role of nuclear medicine in the initial staging, treatment planning, and evaluation of treatment response is highlighted. 1 [18F]F-FDG PET enables accurate delineation of primary tumors, detection of nodal involvement, and identification of distant metastases. This noninvasive approach aids in guiding treatment decisions and evaluating therapeutic response, ultimately improving patient outcomes. The value of [18F]F-FDG PET is evidenced in the adoption of [18F]F-FDG PET/computed tomography (CT) into most oncological societies (European Society for Medical Oncology, National Comprehensive Cancer Network, European Society of Gynaecological Oncology) guidelines pertaining to most of the gynecological malignancies, especially for staging, treatment response assessment, and recurrence assessment. 2 3 4 5 While [18F]F-FDG PET is embedded in cancer care, it is not without its limitations, which are important to note for accurate interpretation of gynecological scans.

Moving beyond the tumor boundaries, novel PET radiopharmaceuticals offer a window into the dynamic interplay within the tumor microenvironment with a promise for improving patient management. Cancer-associated fibroblasts express fibroblast-activated proteins (FAP), which can be imaged using small molecular targets of fibroblast activation protein inhibitors (FAPI) that may be labeled to both Gallium-68 or Flourine-18. 6 7 The emergence of FAPI PET imaging brought huge excitement, as it promised to provide insights into the tumor microenvironment of various malignancies. 8 As more evidence emerged on the potential applications of this tracer, it became apparent that it may not necessarily be the substitute for [18F]F-FDG; however, it has specific indications in which it outperforms [18F]F-FDG. Dendl et al demonstrated the high-tracer uptake and higher tumor-to-background ratios in primary tumors and metastatic lesions in patients with various gynecological malignancies. 9 10 The greatest value of FAPI imaging is its potential to prognosticate and for therapeutic applications. More research is needed in this sphere to determine the best FAPI molecule and the most suited radioisotope that can match the biological half-life resulting in synergism that will have remarkable and sustained therapeutic benefits.

Hypoxia imaging represents another frontier in gynecological oncology, offering valuable information that can aid in precision medicine. Tumor hypoxia results in aggressiveness and treatment resistance. Radionuclide imaging with [18F]F-FDG has been purported to be an indirect marker of hypoxia; however, the evidence for this is lacking, and as such, more hypoxia-specific tracers like [18F]F-fluoromisonidazole ([18F]F-FMISO were assessed to enable noninvasive assessment of tumor hypoxia, guiding the selection of hypoxia-targeted therapies and enhancing treatment efficacy. Over the years adaptations to ^18^ F-FMISO resulted in second- and third-generation ^18^ F-labeled hypoxia tracers [18F]F-FAZA, [18F]F-EF5, [18F]F-FETNIM with improved kinetics. 11 ^68^ Ga-labeled nitroimidazoles were also investigated in preclinical and clinical settings. In women with cervical cancer, it was shown that patients with hypoxia as determined by immunohistochemistry had higher mean standardized uptake values and tumor-to-muscle ratios. 12 Although the study had few patients, this work showed the huge potential for [68Ga]Ga-nitroimidazole imaging, and it calls for larger studies and various other peptides to be pursued.

Additionally, hormonal imaging emerges as a promising avenue for characterizing hormone-sensitive gynecological malignancies, such as estrogen receptor-positive endometrial or ovarian cancers. Radiotracers targeting estrogen receptor α (ERα) offer a noninvasive evaluation of in vivo receptor expression and tumor heterogeneity. This further enhances prognostication of gynecological patients and helps predict as well as monitor response to hormonal/endocrine therapies. The PET tracer ^18^ F-fluro-17β-estradiol [18F]F-FES has been investigated in endometrial and ovarian cancers. 13 14 By providing real-time information about hormone receptor status, hormonal imaging aids in individualizing treatment regimens and optimizing therapeutic outcomes.

Other aspects of the tumor microenvironment including angiogenesis [68Ga]Ga-RGD and chemokine receptor 4 [68Ga]Ga-CXCR4 expression have also been investigated, albeit on a small scale.

The evolution of radionuclide imaging transcends traditional boundaries, paving the way for personalized oncology approaches tailored to the individual patient. Multimodal imaging strategies, combining PET or single-photon emission CT with other imaging modalities like CT, or magnetic resonance imaging offer complementary information regarding tumor morphology, vascularity, and metabolic activity. This integrated approach enhances diagnostic accuracy and facilitates comprehensive tumor characterization. Additionally, advancements in image analysis techniques, including artificial intelligence and radiomics, hold promise for refining risk stratification and personalized treatment strategies in gynecological malignancies.

Radionuclide imaging stands as a cornerstone in the comprehensive management of gynecological malignancies, offering invaluable insights into the tumor, its microenvironment, and beyond. From metabolic imaging for accurate staging to exploring the complexities of the tumor microenvironment through FAPI, hypoxia, and hormonal imaging, radionuclide techniques continue to reshape the landscape of gynecological oncology. As we navigate the complexities of cancer biology, the integration of radionuclide imaging into personalized oncology approaches holds the promise of improved outcomes and enhanced patient care in the fight against gynecological cancers.
